# Is Valproate Depressogenic in Patients Remitting from Acute Mania? Case Series

**DOI:** 10.1155/2015/456830

**Published:** 2015-11-17

**Authors:** Kamini Vasudev, Priya Sharma

**Affiliations:** ^1^Departments of Psychiatry and Medicine, Schulich School of Medicine and Dentistry, Western University, Victoria Hospital, London Health Sciences Centre, A2-513, London, ON, Canada N6A 5W9; ^2^Department of Psychiatry, Schulich School of Medicine and Dentistry, Western University, Victoria Hospital, London Health Sciences Centre, B-8, London, ON, Canada N6A 5W9

## Abstract

Valproate is an effective antimanic agent and is recommended as a first-line medication in the treatment of acute mania. Current evidence based guidelines recommend that valproate should be given as a loading dose as it produces a rapid antimanic and antipsychotic response with minimal side-effects. However, no clear guidelines are available on the appropriate dosing or serum levels of valproate in the continuation or maintenance phase of bipolar disorder. We present 4 clinical cases to hypothesize that the higher doses of valproate, such as those used in the treatment of acute mania, may cause a depressive switch. So consideration should be given to reducing the dose of valproate if a patient develops depressive symptoms following recovery from the manic episode, as a therapeutic strategy. The cases also indicate that relatively lower doses and serum levels of valproate are effective in the maintenance phase compared to those needed in the acute manic phase of bipolar disorder. This is the first set of case series that questions the depressogenic potential of valproate in patients remitting from an acute manic episode. It highlights that different doses and serum levels of valproate may be therapeutic in different phases of bipolar disorder.

## 1. Introduction

Bipolar disorder is a severe mental illness characterized by recurrent episodes of mania and depression and is associated with high morbidity and mortality. Valproate is an anticonvulsant that is commonly used in the treatment of bipolar disorder (BPD). It has been found to be effective in the treatment of acute mania and is now one of the first-line agents [1–3]. There is limited evidence to confirm its role in the treatment of an acute depressive episode [4–6]. The efficacy of valproate has been found to be comparable to lithium in the maintenance phase of BPD based on results from a few randomized controlled trials [7–10].

In acute mania, valproate oral loading (doses of 20–30 mg/Kg body weight) produces a rapid antimanic and antipsychotic response with minimal side-effects [11–13]. Additionally, there seems to be a linear relationship between valproate serum concentration and response in acute mania. Serum levels between 50 and 100 *μ*g/mL have been found to be therapeutic with the target blood level of valproate for optimal response in acute mania being above 94 *μ*g/mL [14, 15].

The evidence available on either the dosing range or target serum levels of valproate in the continuation or maintenance phase of BPD is poor [16].

We present 4 cases to hypothesize that (a) higher doses of valproate, such as those used in acute mania, may induce a depressive switch following remission from acute mania and (b) relatively lower doses and serum levels of valproate are needed to be effective in the maintenance phase as compared to the manic phase of BPD.

## 2. Case 1

Case 1 is a 57-year-old woman (weight = 77 Kg) who was admitted to hospital with an acute manic episode. She had a previous history suggestive of a depressive episode, for which she was prescribed escitalopram 10 mg by her family physician about 2 months prior to current admission. At admission, escitalopram was discontinued and divalproex sodium EC 500 mg daily was initiated, the dose of which was increased to 1000 mg daily at bedtime within a week. The steady state serum level was 452 mmol/litre (therapeutic range 350–700). Risperidone (1.5 mg hs) was added to address some unresolved paranoid symptoms. The patient improved clinically enough in 4 weeks to be discharged from hospital. During a subsequent ambulatory appointment one month later, she presented with depressive symptoms including a lack of motivation, low energy, and tearfulness. She complained of “grogginess” with risperidone; henceforth the dose was tapered to 1 mg at bedtime and then 0.5 mg after another month. Three months after her discharge she continued to complain about ongoing depressive symptoms. Her serum valproate level on the 1000 mg dose was 784 mmol/L. Her dose of valproate was then reduced down to 750 mg at bedtime. At her next presentation in six weeks, she was assessed to be euthymic and the serum level of valproate was 362 mmol/L. She continued to remain stable for subsequent 6 months and then experienced another depressive episode. At that point, her serum valproate level was 528 mmol/L whilst on a valproate dose of 750 mg. She was also continuing risperidone 0.5 mg at bedtime. Escitalopram 10 mg was added for management of symptoms of depression and risperidone was discontinued for feelings of “fogginess” on the medication. At the time of reporting the case, the patient has been in remission for about three years on a combination of valproate 750 mg and escitalopram 10 mg.

## 3. Case 2

Case 2 is a 40-year-old woman (weight = 59 Kg) who was admitted with an acute manic episode. She had been discharged from hospital 4 weeks prior to this admission, when she was admitted with a manic episode and discharged on risperidone 1 mg at bedtime, which she soon stopped taking due to side-effects. At this admission, divalproex sodium EC was started at 250 mg at bedtime and titrated up to 1000 mg. Her steady state serum valproate level was 652 mmol/L. She improved in mental state and was discharged from hospital in three weeks. At follow-up visit at two weeks, she complained of feeling sedated on valproate and so the dose was reduced to 750 mg and then to 500 mg 6 weeks later. She remained stable in her mood for 5 months but then reported that “valproate was making her slower and lower in her mood.” She reported that she had reduced the dose of valproate further down to 62.5 mg (half of 125 mg pill) and had added low dose of aripiprazole (5 mg) in consultation with her family physician, to help with low mood. She remains in remission on this combination for about three years at the time of reporting the case.

## 4. Case 3

Case 3 is a 32-year-old man (weight = 89 Kg) who presented with the first episode of mania in the summer of 2011, without any psychiatric history. His past medical history was significant for a car accident, which had occurred 17 years priorly. He had been in a coma for three days following the collision, but CT head was unremarkable. For the manic episode, he was treated with divalproex sodium EC at a dose of 1000 mg at bedtime as well as Seroquel XR 200 mg. The steady state serum valproate level was 663 mmol/L. He improved in mental state and was discharged from hospital in 3 weeks on the same dose of valproate, but his Seroquel was discontinued due to feelings of grogginess. At the outpatient appointment one month following discharge, he presented with low mood and a decision was made to decrease the valproate to 750 mg at bedtime. The steady state serum level at this point was 425 mmol/L. A month later, the patient had reduced the dose on his own to 500 mg due to feeling depressed and felt that reducing the dose had helped. His valproate level was measured at 168 mmol/L. He remained stable for roughly two months before returning to clinic, feeling depressed and anxious. Escitalopram 10 mg daily and clonazepam 0.5 mg daily prn were started for these symptoms. He did not continue on escitalopram, as he would often forget to take it and reported improvement with clonazepam alone. He remained stable on valproate 500 mg and clonazepam for 18 months. The dose of valproate was then increased to 625 mg for better control of anxiety symptoms. He remains stable on this dose for about two and a half years, as well as 0.5 mg of clonazepam and trazodone 50 mg for sleep at the time of reporting this case.

## 5. Case 4

Case 4 is a 42-year-old woman (weight = 59 Kg). She was admitted with an acute manic episode with psychosis. She had been previously diagnosed with major depressive disorder with psychosis and had been stable on escitalopram 20 mg for about a year. This was the first manic episode precipitated by work related stressors. The escitalopram was discontinued and the patient was initiated on divalproex sodium EC, the dose of which was increased to 1000 mg over 5 days. In addition she was also started on risperidone 0.5 mg and the dose increased to 1.5 mg at bedtime over one week. The serum level of valproate reported after one week was 1032 mmol/L and so the dose was reduced from 1000 mg to 750 mg at bedtime. The serum valproate level after one week was 877 mmol/lit. The dose was further reduced to 500 mg and serum level after one week was 644 mmol/L. The complete blood count and liver function tests were normal.

The serum prolactin level after one week of risperidone 1.5 mg dose was found at 146 *μ*gm/L. She started expressing delusion of pregnancy. A decision was made to cross-taper risperidone with olanzapine 10 mg hs. Serum prolactin came down to 36 *μ*gm/L in two weeks.

Her manic and psychotic symptoms resolved by week 3 of admission; however, she reported feeling depressed, being tearful and guilty, and having difficulty in making decisions by week 2. As the dose and serum levels of valproate went down, her mood started improving and she was eventually discharged after 5 weeks of admission. She was on valproate 500 mg at bedtime and olanzapine 10 mg at bedtime at discharge. At outpatient follow-up in two weeks, she continued to experience some residual depressive symptoms and so valproate was reduced further down to 375 mg and olanzapine continued at 10 mg at bedtime. The dose of olanzapine was gradually reduced to 5 mg due to complaints of excessive sedation. She remains euthymic for 12 months at the time of reporting this case; current medications include valproate 375 mg and olanzapine 5 mg daily.

## 6. Discussion

All the four cases presented above were diagnosed with a manic episode, at the time of admission to hospital. Case 1 may be different from the remaining cases as her index manic episode appears to be induced by the use of an antidepressant. It is possible that she initially presented with a mixed episode which may have been misdiagnosed as depressive episode by the family physician and treated with escitalopram; by the time she was admitted to hospital, the clinical picture was predominantly manic.

We observe from all of the four cases that even though the dose of valproate was reduced in the maintenance phase of BPD, there was no recurrence of mania in the three-year follow-up period, in spite of adding a low dose of antidepressant in Case 1 for a significant period of time. Case 2 received additional aripiprazole, which may have antimanic effects, but in her case it was prescribed for treating depressive symptoms. Therefore, we hypothesize that relatively lower doses of valproate are required to prevent manic recurrence as compared to doses used for treatment of an acute manic episode. As such, using a lower dose may be of benefit as it reduces the risk of dose related adverse effects during the maintenance phase of BPD.

Another observation made from these cases is that the patients who were continued on the same doses of valproate which were effective for acute mania (doses of ≥750 mg daily), in the continuation phase, went on to develop prominent symptoms of depression. It is possible that this was the natural course of illness and valproate was not able to prevent a relapse of depressive episode; however, this seems unlikely as the depressive symptoms improved as the dose of valproate was reduced. It is therefore additionally hypothesized that the higher dose of valproate used for acute mania may induce a depressive switch and that reducing the dose of valproate may be of benefit in patients who develop depressive symptoms following recovery from a manic episode. This may preclude the need for additional psychotherapeutic agent for the treatment of the depressive episode, as well as reducing the risk of adverse effects.

We conducted a comprehensive literature search to answer two questions raised from our case series: (a) What doses or serum levels of valproate are efficacious and safe in the continuation or maintenance phase of BPD and (b) is valproate “depressogenic” or does it induce depressive switch in patients with BPD?

Our search revealed no published prospective studies examining the relationship between dosing or serum levels of valproate and its efficacy and safety in the continuation or maintenance phase of BPD. A recent updated Cochrane review on valproate in the maintenance treatment of BPD by Cipriani et al., 2013 [7], identified six randomized controlled trials (*n* = 876 participants) lasting 6 to 24 months. Unfortunately, none of the trials included in this Cochrane review specifically examined either the doses or serum levels of valproate in the maintenance treatment of BPD. In fact, the trials used the doses as recommended for acute mania (750 to 1250 mg daily) and aimed to achieve serum levels as indicated for a manic episode (>50 *μ*g/mL) [8–10].

There is one study that looked at the valproate serum levels in maintenance therapy of BPD [16]. The authors retrospectively examined the serum valproate levels in 17 bipolar I and 24 bipolar II disorder outpatients who had been treated with stable doses of valproate successfully for at least 12 months as prophylactic therapy. The trough serum valproate levels were 52.2 ± 20.4 microg/mL in bipolar I and 41.0 ± 18.3 microg/mL in bipolar II patients, respectively. The authors suggested that there may be a correlation between the level of valproate required for stabilization and the subtype of the BPD.

None of the trials conducted on use of valproate in BPD so far have reported or examined its potential to induce a depressive switch. It is important to note that the evidence for its antidepressant efficacy is generally poor and valproate monotherapy is not recommended as a first-line therapy for the treatment of bipolar I or bipolar II depressive episode [1, 5, 6]. The BALANCE trial looked at lithium plus valproate combination therapy versus monotherapy for relapse prevention in bipolar I disorder. On secondary outcome analysis, this trial indicated that advantage of lithium compared to valproate was most apparent for depressive relapses [9].

It is interesting to note that, according to the latest CANMAT guidelines released in 2013, valproate monotherapy is recommended as one of the first-line agents in the maintenance phase of bipolar I disorder but only as second-line agent for maintenance phase of bipolar II disorder [1]. Does this mean valproate may have differential efficacy in the maintenance phase of bipolar I and bipolar II disorders?

## 7. Conclusion

From our case series, it is suggested that the higher doses of valproate, such as those used in the treatment of acute mania, may cause a depressive switch following remission from the manic episode. So consideration should be given to reducing the dose of valproate if a patient develops depressive symptoms following recovery from the manic episode, as a therapeutic strategy. The cases also indicate that relatively lower doses and serum levels of valproate are effective in the maintenance phase compared to those needed in the acute manic phase of bipolar disorder. We acknowledge the limitations of the above hypotheses as these are based on the observations made in the above reported four cases only.

Further good quality research, including randomized controlled trials, is required to examine the minimum effective doses (and serum levels) of valproate in the maintenance therapy of BPD as well as to examine its potential to induce a depressive switch following remission from acute mania. The differential effect of valproate in bipolar I and bipolar II disorder with and without rapid cycling/mixed features also needs to be explored further.
